# The Cystoscope Sheath as a Platform for Performing Retrograde Intrarenal Surgery in a Transplanted Kidney with Complex Renal Anatomy

**DOI:** 10.1089/cren.2016.0056

**Published:** 2016-05-01

**Authors:** Aaron F. Brafman, Marla J. Wardenburg, Vincent Bird

**Affiliations:** Department of Urology, UF Health/College of Medicine, Gainesville, Florida.

## Abstract

***Background:*** Endourology is a widely used means by which to manage urolithiasis. Patient anatomy can oftentimes limit what can be accomplished with current technology.

***Case Presentation:*** This is a case of a patient with renal and ureteral stones within a transplant kidney. Her anatomy would not allow for a standard retrograde ureteroscopic approach. We describe a method by which to overcome this difficult scenario by using a rigid cystoscope as a platform by which a ureteroscope was passed to allow for stone removal.

***Conclusion:*** For this difficult case, we effectively used our instruments to achieve our goal of retrograde ureteroscopy in a transplant kidney with an unfavorably angulated ureter.

## Introduction and Background

Upper urinary tract endoscopy has long been established as a safe and efficient means of managing urolithiasis with great success. With technological advances, ureteroscopy has evolved into a powerful tool in the armamentarium of the urologist. Management of patients with complex anatomy is now possible while avoiding more invasive interventions such as percutaneous nephrostolithotomy and open/laparoscopic surgery.^1^ The following is a case report illustrating the use of current technology to overcome a difficult intraoperative scenario in the management of urolithiasis.

## Presentation of Case

This is a case of a 71-year-old African American female with a history of cryptogenic liver cirrhosis who underwent a combined liver–kidney transplant in 2012. Two years later, she was found to have a transplant ureteral stone and an 8 mm upper pole nonobstructing renal stone. She was taken to the operating room for ureteroscopic management of her stone burden. Her case was complicated by unfavorable transplant renal anatomy. A percutaneous approach was not possible given small bowel overlying the anterior surface of the transplant kidney.

**Fig. 1.**
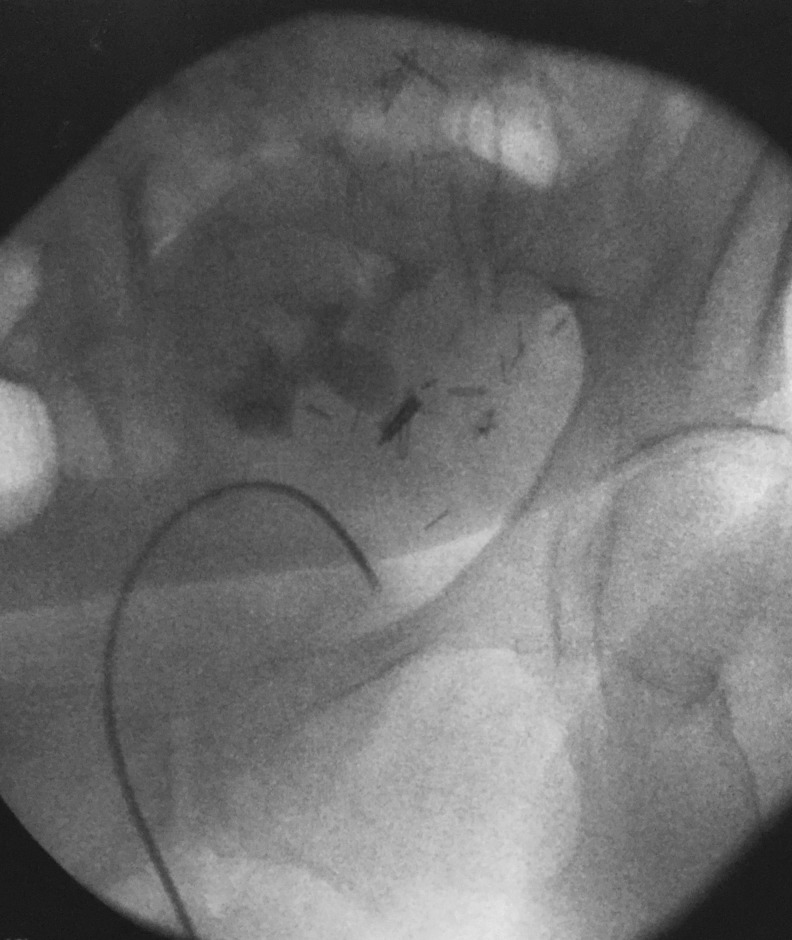
Backward deflection of flexible ureteroscope into the bladder.

**Fig. 2.**
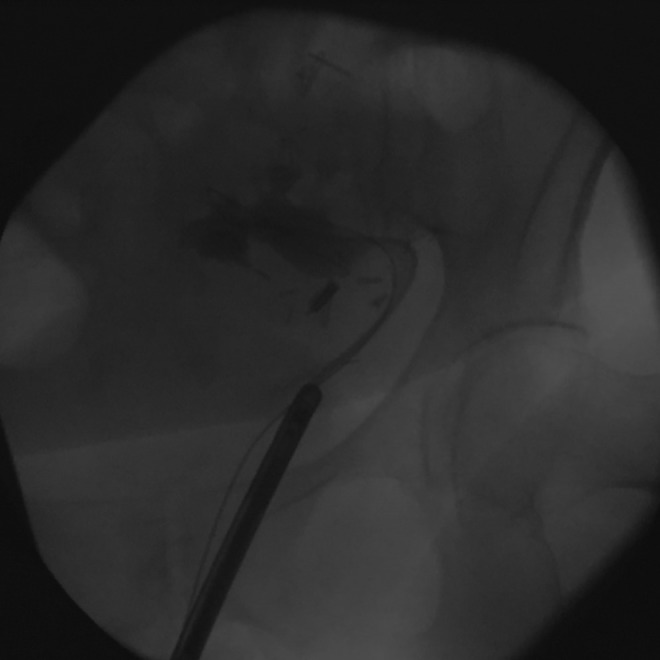
The cytoscope sheath is positioned at the transplanted ureteral orifice, allowing for passage of the flexible ureteroscope into the kidney.

**Fig. 3.**
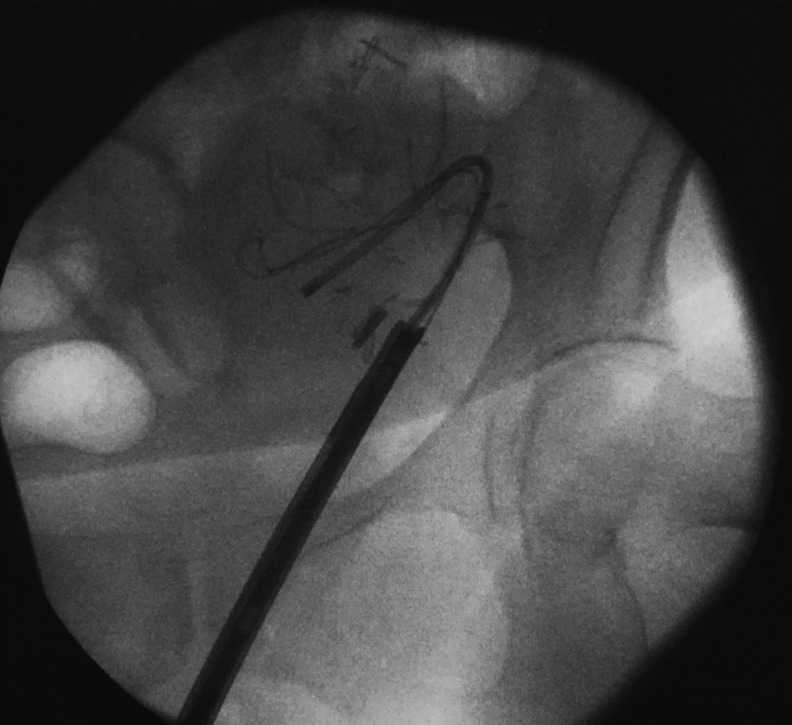
Another view of the cytoscope sheath positioned at the transplanted ureteral orifice, allowing for passage of the flexible ureteroscope into the kidney.

The ureteroneocystotomy was identified at the left anterior bladder wall, facing the caudad at an angle, and was eventually cannulated. Retrograde pyelography revealed an ∼90° angulation of the mid-ureter toward the renal pelvis seen medially. Access was established with both a standard teflon wire and a superstiff teflon guidewire. Due to the location of the ureteroneocystotomy as well as the ureteral angulation, a flexible ureteroscope could not be advanced beyond the midureter, resulting in deflection of the ureteroscope into the urinary bladder (Fig. 1). To overcome this, we positioned a rigid cystoscope at the level of the transplant ureteral orifice. While one operator held the cystoscope in position, a second endoscopist advanced the flexible ureteroscope through the cystoscope's working channel over the guidance of a superstiff wire and into the transplant kidney. The rigid cystoscope acted as an advanced platform and provided a support for antegrade passage of the flexible ureteroscope, acting as a backboard preventing backward slippage. Laser lithotripsy and subsequent ureteral stent placement were then effectively performed in the standard manner. The cystoscope was kept in the aforementioned position throughout the duration of the operation to negotiate the ureteroscope into each renal calix while avoiding deflection into the ureter and bladder (Figs. 2, 3).

Because of the inability to place a ureteral access sheath, the stone was fragmented to dust so no fragments were retrieved for crystallographic analysis. The patient was seen in follow-up with no radiographic evidence of residual stone burden. A 24-hour urine collection was obtained, and she is currently being effectively managed with dietary modification.

## Discussion and Literature Review

Ureteroscopy performed in those with native anatomy is possible, in part, from the orthotopic location of the ureteral orifices, allowing the urologist to take advantage of the bladder trigone as a backbone to advance wires and ureteroscopes. The purchase at the level of the ureteral orifice is lost in cases as in that described previously, where the non-native ureter is in a more anterior location. The use of larger, more rigid scopes as a platform to launch a second device is well established and utilized in other disciplines. Endoscopic retrograde cholangiopancreatography is a procedure that applies upper gastrointestinal endoscopy to manage pathology of the bile and pancreatic ducts. Smaller devices are passed through working channels of the larger endoscope to instrument the smaller caliber biliary tract.^2^ Passage of smaller scopes through a larger scope has also been described in the field of urology. The first flexible ureteroscope, described by Marshall in 1964, had no method of deflecting the tip; it was passed through a 26F rigid cystoscope into the distal ureter, allowing for ureteral stone extraction.^3^ Graversen and colleagues described passage of a ureteroscope through the working channel of a nephroscope during percutaneous nephrolithotomy in cases with narrow infundibuli and poor observation.^4^

## Conclusion

For this difficult case, we effectively used our instruments to achieve our goal of retrograde ureteroscopy in a transplant kidney with an unfavorably angulated ureter. Often the flexible ureteroscope will pass to the transplant kidney without this technique, but if insertion is angulated in an unfavorable direction, we will lose the needed force vector without the advanced platform.
